# The prognostic significance of long noncoding RNAs in bladder cancer: A meta-analysis

**DOI:** 10.1371/journal.pone.0198602

**Published:** 2018-06-05

**Authors:** Yuexin Xia, Zhiyuan Liu, Weijian Yu, Shihang Zhou, Linnan Shao, Wenqian Song, Ming Liu

**Affiliations:** 1 Dalian Blood Center, Dalian, Liaoning, China; 2 Biomedical Business Department, Panasonic Appliances Cold Chain (Dalian)co., Ltd, Dalian, Liaoning, China; 3 Department of Cell Biology, Dalian Medical University, Dalian, Liaoning, China; National Center for Biotechnology Information, UNITED STATES

## Abstract

**Introduction:**

Bladder cancer (BC) is one of the most common urologic malignancies and it is urgently needed to identify novel potential prognostic biomarkers for predicting prognosis and progression of patients with BC in clinical practice. Previous research has revealed that long noncoding RNAs (lncRNAs) played critical roles in BC, and may serve as novel potential prognostic biomarkers in patients with BC. Therefore, we conducted this meta-analysis to clarify the prognostic potential of lncRNAs in BC patients.

**Methods:**

A comprehensive search was performed in PubMed, Web of Science, and China National Knowledge Infrastructure (CNKI). According to the predefined exclusion and inclusion criteria, a total of 9 recently published articles comprising 13 lncRNAs and 666 BC patients were included into this meta-analysis. We analyzed the hazard ratios (HRs) and 95% confidence intervals (CIs) to determine the relationship between lncRNAs expression and survival outcomes. We also analyzed the odds ratio (ORs) and 95% confidence intervals (CIs) to assess the association between lncRNAs expression and clinicopathological characteristics, including histological grade, gender, multifocality, tumor size, and tumor stage.

**Results:**

Our results revealed that high lncRNAs expression was associated with shorter overall survival in Asian BC patients (pooled HR = 2.32, 95% CI: 1.35–4.00, *P* = 0.002, random-effect). High lncRNAs expression levels were significantly associated with histological grade (G2-G3 vs. G1: OR = 3.857, 95%CI: 1.293–11.502, *P* = 0.015, random-effect).

**Conclusions:**

In summary, this meta-analysis has demonstrated that lncRNAs could be used as potential prognostic markers for BC and high lncRNAs expression could predict poor prognosis among Asian BC patients.

## Introduction

Bladder cancer (BC) is one of the most common urologic malignancies, with nearly 430, 000 new cases diagnosed in 2012 worldwide [1]. Overall, 75% of the patients with BC are categorized as non-muscle-invasive bladder cancer (NMIBC) [2], which is associated with a high risk of recurrence and may progress to muscle invasive bladder cancer (MIBC) [3]. MIBC is associated with poor prognosis and the estimated 5-year survival rate remains at only 50% [4]. As a consequence, it’s crucial to identify novel potential prognostic biomarkers for predicting prognosis and progression of patients with BC in clinical practice.

Long non-coding RNAs (lncRNAs) are a class of non-protein-coding RNA molecules with more than 200 nucleotides [5]. It is reported that lncRNAs play critical roles in various cell biological processes, such as cellular differentiation, gene expression, protein localization, and DNA damage response [6]. An increasing number of studies have revealed that lncRNAs played tremendous roles in various human diseases, including cancer [7, 8]. More importantly, aberrant expression of multiple lncRNAs were found to be involved in the tumorigenesis and many of them were correlated with cancer prognosis [9–11]. Multiple lncRNAs have been reported to be promising prognostic indicators for cancers, such as hepatocellular carcinoma [12], non small cell lung cancer [13, 14], osteosarcoma [15], ovarian carcinoma [16], and renal cell carcinoma [17]. So far, many studies have shown that lncRNAs also played critical roles in BC [18], these findings support that lncRNAs can be developed as novel potential prognostic biomarkers in patients with BC.

However, owing to the limitations in sample size, single study may be inaccurate and insufficient. Thus, studies should be analyzed systematically to uncover the potential prognostic value of lncRNAs in patients with BC. Nevertheless, no meta-analysis has been carried out to provide a precise estimation. As a consequence, we conducted this meta-analysis to explore the prognostic value of lncRNAs and the association between lncRNAs and clinicopathological characteristics by combined analysis of data from the published articles.

## Materials and methods

### Search strategies

The contents of this review are in accordance with the standard guidelines of Preferred Reporting Items for Systematic Reviews and Meta-analysis (PRISMA) (S1 Checklist) [19]. We searched the databases PubMed, Web of Science, and China National Knowledge Infrastructure (CNKI) for relevant literatures about the prognostic value of lncRNA in BC. The search was performed by both text word and MeSH terms to increase the sensitivity. The following search terms were used: (“RNA, Long Noncoding”, “lncRNA”, “long noncoding RNA”, “Long intergenic non-coding RNA”) AND (“Urinary Bladder Neoplasms”, “Bladder Neoplasm”, “Bladder Tumor”, “Urinary Bladder Cancer”, “Bladder Cancer”) AND (“Prognosis”, “Prognostic”, “outcome”, “survival”, “recurrence”, “recurrence”). Additionally, manual searches were performed using the reference lists of the relevant articles to identify potentially eligible literatures. The retrieval time was from inception to May 2017.

### Inclusion and exclusion criteria

The inclusion criteria were as follows: (1) studies evaluated the association between lncRNA(s) expression and prognosis of bladder cancer; (2) the survival outcomes were measured with overall survival (OS) or recurrence-free survival (RFS); (3) sufficient data were provided to estimate hazard ratios (HRs) and their 95% confidence interval (95% CI). The exclusion criteria were as follows: (1) insufficient data for HR and 95% CI estimation; (2) reviews, letters, or laboratory articles; (3) sample cases fewer than 30.

### Data extraction

Data was carefully retrieved by two investigators (Yuexin Xia and Zhiyuan Liu) independently. The following information was extracted from each study: (1) publication information: the surname of first author and the year of publication; (2) patients’ characteristic information: study population, sample size, and follow-up duration; (3) lncRNA information: detection methods, survival results, and cut-off definition; (4) HRs and corresponding 95% CIs for survival analysis. The study quality was assessed in accordance with the Newcastle-Ottawa Scale (NOS) [20].

## Statistical analysis

For the prognostic meta-analysis, HRs and corresponding 95% CIs were used to assess the relationship between lncRNAs expression and its prognostic value in BC. HRs and corresponding 95% CIs were extracted directly from data in included studies or calculated with available data by the method from Parmar. et al [21]. An observed HR>1 implied a poor prognosis. ORs and corresponding 95% CIs were used to evaluate the association between lncRNAs expression and clinical characteristics. A OR>1 implied that high levels of lncRNA was associated with parameter.

The statistical significance of the pooled HRs and ORs were determined using Z-test; a *P* value < 0.05 was considered statistically significant. Heterogeneity was evaluated by Q and *I*^*2*^ tests. If the heterogeneity was not significant (*I*^*2*^ < 50%, *P* value > 0.05), the fixed-effects model was used. Otherwise, a random-effects model was used (*I*^*2*^ ≥ 50%, *P* value ≤ 0.05).

Publication bias and sensitivity analysis were performed to test the effect of an individual study on pooled HR and OR. For publication bias assessing, Begg’s funnel plot and Egger’s regression test were employed. An asymmetric plot and the *P* value < 0.05 were considered a significant publication bias.

All of the statistical analyses were performed by using STATA12.0 software package (Stata Corporation).

## Results

### Study selection and characteristics

**Fig 1:**
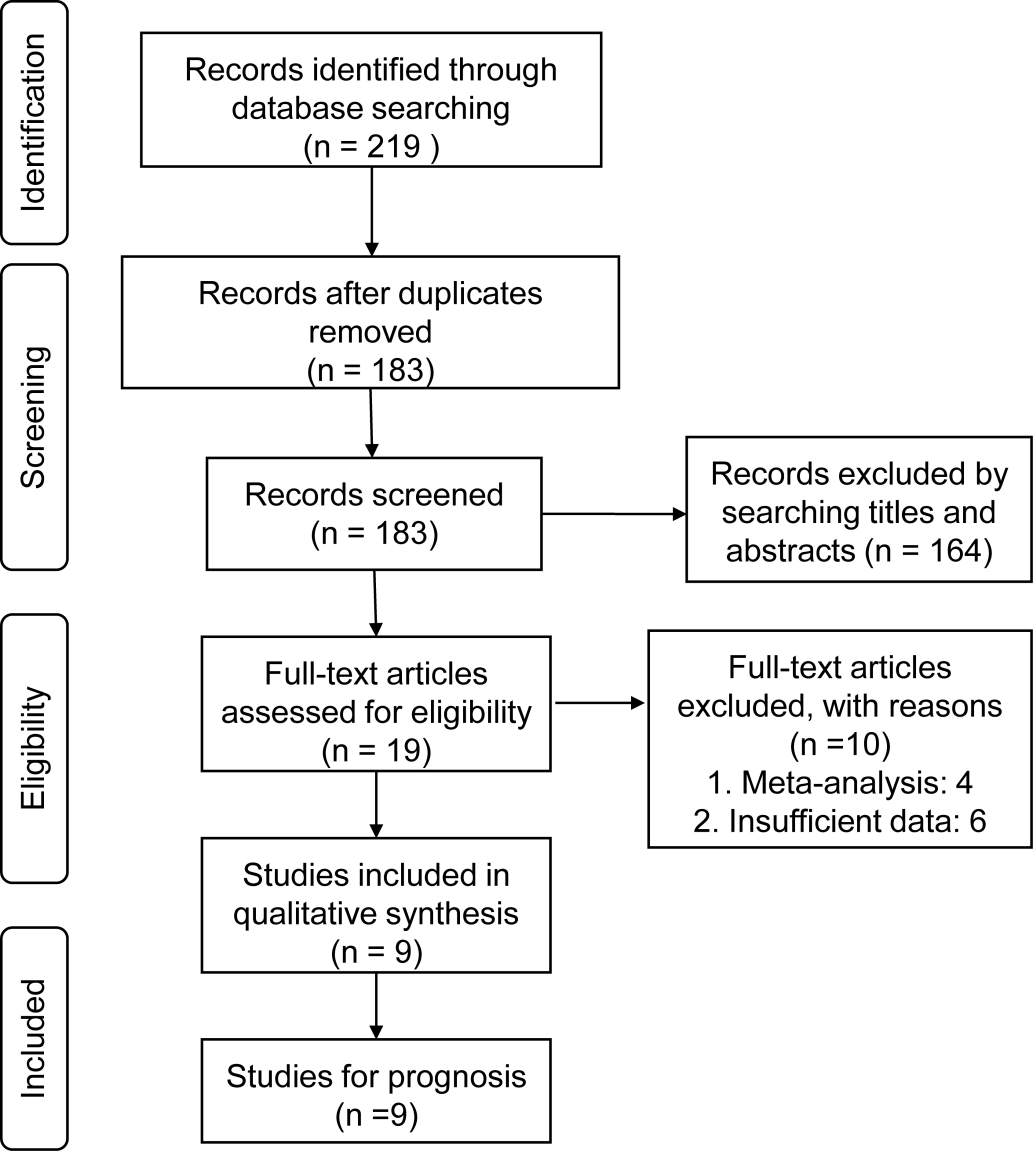
Flow chart of literature selection process in the meta-analysis.

According to the predefined criteria, a total of 9 eligible studies were acquired from 3 databases, including PubMed, Web of Science, and CNKI [22–30]. Fig 1 shows the literature inclusion procedure. The details of the studies included in the meta-analysis are shown in Table 1.

**Table 1:** Characteristics of studies included in the meta-analysis.

Study	lncRNAs	Country	Follow-up	Cut-off	Method	Sample	Outcome	HR(95% CI)	HR	NOS
			(month)			size		High/Low	estimate	score
Droop 2017[22]	UCA1	Germany	22.7(0.2–198)	Median	qRT-PCR	106	OS	0.576(0.367–0.876)	Reported	6
Droop 2017[22]	Linc-UBC1	Germany	22.7(0.2–198)	Median	qRT-PCR	106	OS	0.953(0.619–1.468)	Reported	6
Droop 2017[22]	TUG1	Germany	22.7(0.2–198)	Median	qRT-PCR	106	OS	0.579(0.375–0.895)	Reported	6
Droop 2017[22]	ncRAN	Germany	22.7(0.2–198)	Median	qRT-PCR	106	OS	0.416(0.543–1.287)	Reported	6
Droop 2017[22]	MALAT1	Germany	22.7(0.2–198)	Median	qRT-PCR	106	OS	0.547(0.353–0.848)	Reported	6
Droop 2017[22]	H19	Germany	22.7(0.2–198)	Median	qRT-PCR	106	OS	0.904(0.587–1.390)	Reported	6
Droop 2017[22]	GAS5	Germany	22.7(0.2–198)	Median	qRT-PCR	106	OS	0.707(0.459–1.091)	Reported	6
Zhang 2016[23]	UNMIBC	China	36(10–69)	FC>1.5	qRT-PCR	75	RFS	2.362(1.504–4.837)	Reported	7
Duan 2016[24]	MEG3	China	57(4–76)	Median	qRT-PCR	59	RFS	2.222(1.013–4.878)	Reported	6
Duan 2016[24]	SNHG16	China	57(4–76)	Median	qRT-PCR	59	RFS	0.613(0.286–1.312)	Reported	6
Duan 2016[24]	MALAT1	China	57(4–76)	Median	qRT-PCR	59	RFS	1.631(0.756–3.521)	Reported	6
Iliev 2016[25]	TUG1	Czech Republic	30(12–104)	ROC	qRT-PCR	47	OS	2.54 (1.13–5.74)	Reported	8
Zhao 2015[26]	SPRY4-IT1	China	60(Total)	Median	qRT-PCR	68	OS	3.716(2.084–6.719)	Reported	7
Martínez-Fernández 2015[27]	HOTAIR	Spain	28(1–36)	Median	qRT-PCR	64	OS	2.21(1.02–4.81)	Survival curve	6
Chen 2015[28]	n336928	China	60(Total)	Median	qRT-PCR	98	OS	2.377(1.007–5.610)	Reported	8
Tan 2015[29]	TUG1	China	60(Total)	NA	qRT-PCR	54	OS	2.84(1.21–7.20)	Survival curve	6
Fan 2014[30]	MALAT1	China	30(Total)	Median	qRT-PCR	95	OS	1.26(0.68–2.13)	Reported	8

FC = fold change; ROC = receiver operating characteristic; OS = overall survival; RFS = recurrence-free survival; HR: hazard ratio; 95% CI: confidence intervals; UCA1 = urothelial carcinoma associated 1 RNA = ; Linc-UBC1 = Up-regulated in bladder cancer 1; H19 = long noncoding RNA 19; GAS5 = growth arrest-specific 5; UNMIBC = Up-regulated in MIBC; TUG1 = taurine upregulated gene 1; MEG3 = maternally expressed gene 3; SNHG16 = small nucleolar RNA host gene 16; Malat1 = metastasis associated lung adenocarcinoma transcript 1; SPRY4-IT1 = SPRY4 intronic transcript 1; HOTAIR = HOX antisense intergenic RNA.

### Association between lncRNAs expression and OS

**Fig 2:**
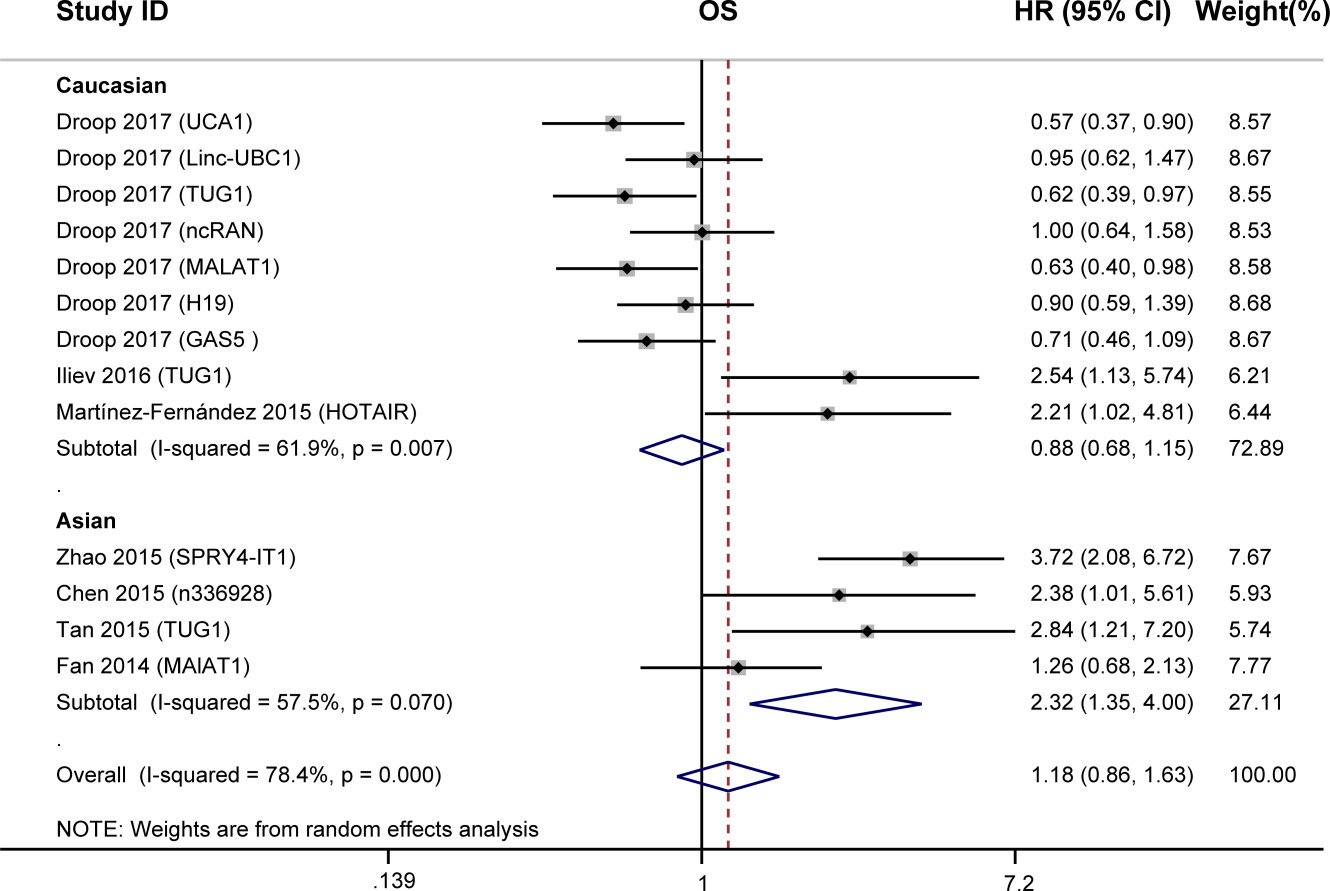
Forest plots for the association between lncRNAs expression and OS of BC patients.

We conducted meta-analysis to investigate the prognostic value of lncRNAs in OS of 532 BC patients from the seven studies. Statistical analyses showed no significant association between the expression of lncRNAs and OS of BC patients (HR = 1.18, 95% CI: 0.86–1.63, *P* = 0.310, random-effects; Fig 2), while a significant heterogeneity existed between studies(*I*^*2*^ = 78.4%, *P* = 0.000).

Due to the presence of obvious heterogeneity, we performed subgroup analyses based on the ethnicity, follow-up period, and the expression level of lncRNAs in BC patients. Subgroup analysis by ethnicity indicated that high lncRNAs expression was associated with shorter overall survival in Asian BC patients (HR = 2.32, 95% CI: 1.35–4.00, *P* = 0.002, Fig 2) but not in Caucasians (HR = 0.88; 95% CI: 0.68–1.15, *P* = 0.358). And the heterogeneity decreased from 78.4% to 57.5% and 61.9%, respectively. When grouped according to the follow-up period, the association between high lncRNAs expression and poor OS was found only for studies of shorter follow-up period (≤60 months) (HR = 2.29, 95% CI: 1.50–3.51, *P*<0.001, Table 2). When grouped according to the expression level of lncRNAs in BC patients, there were no association between lncRNAs expression and OS (Table 2).

**Table 2:** Main results of subgroup analyses.

Categories	Subgroups	*n*	HR (95% CI)	*P*	Heterogeneity	
					*I*^*2*^ (%)	*Ph*
All		13	1.18(0.86–1.63)	0.310	78.40	0.00
Ethnicity	Asian	4	2.33(1.35–4.00)	**0.002**	57.50	0.07
	Caucasians	9	0.88(0.68–1.15)	0.358	61.90	0.01
Follow-up	≤ 60	5	2.29(1.50–3.51)	<**0.001**	43.40	0.10
	> 60	8	0.81(0.64–1.03)	0.090	51.90	0.06
Expression level	Increased in tumors	12	1.26(0.89–1.77)	0.190	78.40	0.00

### Association between lncRNAs expression and RFS

**Fig 3:**
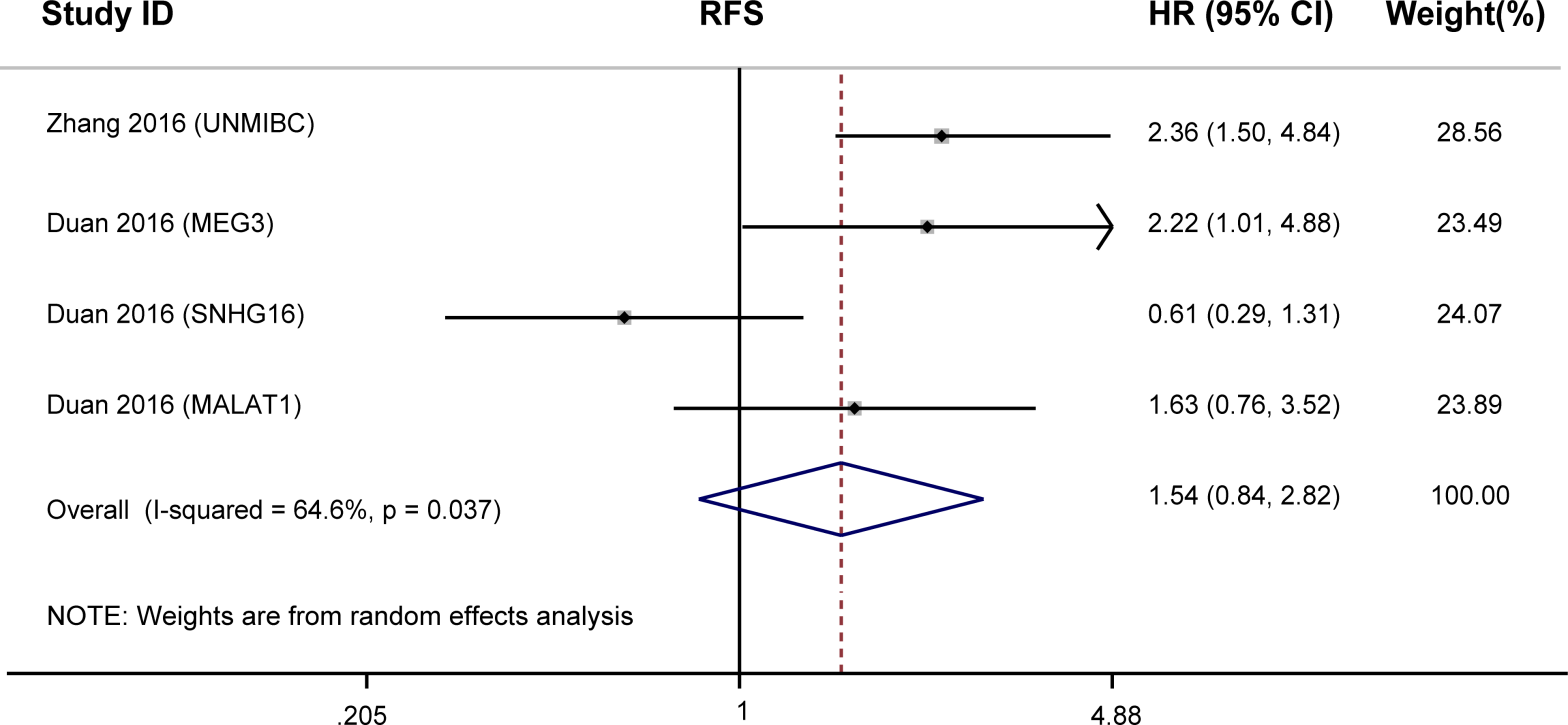
Forest plots for the association between lncRNAs expression and RFS of BC patients.

The prognostic value of lncRNAs in RFS was evaluated in two studies with 134 patients. lncRNAs expression were not significantly associated with RFS (HR = 1.54, 95%CI: 0.84–2.82, *P* = 0.162, random-effects; Fig 3), while a significant heterogeneity existed between studies (*I*^*2*^ = 64.6%, *P* = 0.037). Meta regression analysis, sensitivity analysis, and assessment of publication bias were not performed due to the limited number of included articles.

### Correlation of lncRNAs with clinicopathological characteristics of BC

We conducted a meta-analysis to evaluate the association between lncRNAs expression and clinical characteristics in BC patients. High lncRNAs expression levels were significantly associated with histological grade (G2-G3 vs. G1: OR = 3.857, 95%CI: 1.293–11.502, *P* = 0.015, random-effect), while a significant heterogeneity existed between studies (*I*^*2*^ = 70.2%, *P* = 0.035) (Table 3). Unfortunately, no significant correlation was found with gender (male vs. female: OR = 1.291, 95%CI: 0.782–2.129, *P* = 0.318, fixed-effect), multifocality (multifocal vs. unifocal: OR = 1.109, 95%CI: 0.660–1.861, *P* = 0.696, fixed-effect), tumor size (>3cm vs. ≤3cm: OR = 0.964, 95%CI: 0.519–1.790, *P* = 0.907, fixed-effect), and tumor stage (Ta,T1 vs. T2-T4: OR = 0.502, 95%CI: 0.199–1.265, *P* = 0.144, random-effect).

**Table 3:** Association between high levels of lncRNAs and clinicopathological characteristics of patients with BC.

Subgroup factor	Studies	Case number	Pooled OR(95% CI)	*P*	Heterogeneity		References
					*I*^*2*^	*P*_*h*_	
Gender (male vs. female)	4	336	1.291(0.782–2.129)	0.318	24.6	0.264	[23], [26], [28], [30]
Multifocality (multifocal vs. unifocal)	3	241	1.109(0.660–1.861)	0.696	47.3	0.15	[23], [26], [28]
Tumor size (>3cm vs. ≦3cm)	2	193	0.964(0.519–1.790)	0.907	0.0	0.494	[28], [30]
Histological grade (G2-G3 vs. G1)	3	261	3.857(1.293–11.502)	**0.015**	70.2	0.035	[26], [28], [30]
Tumor stage (Ta,T1 vs. T2-T4)	2	163	0.502(0.199–1.265)	0.144	50	0.157	[26], [30]

### Publication bias

**Fig 4:**
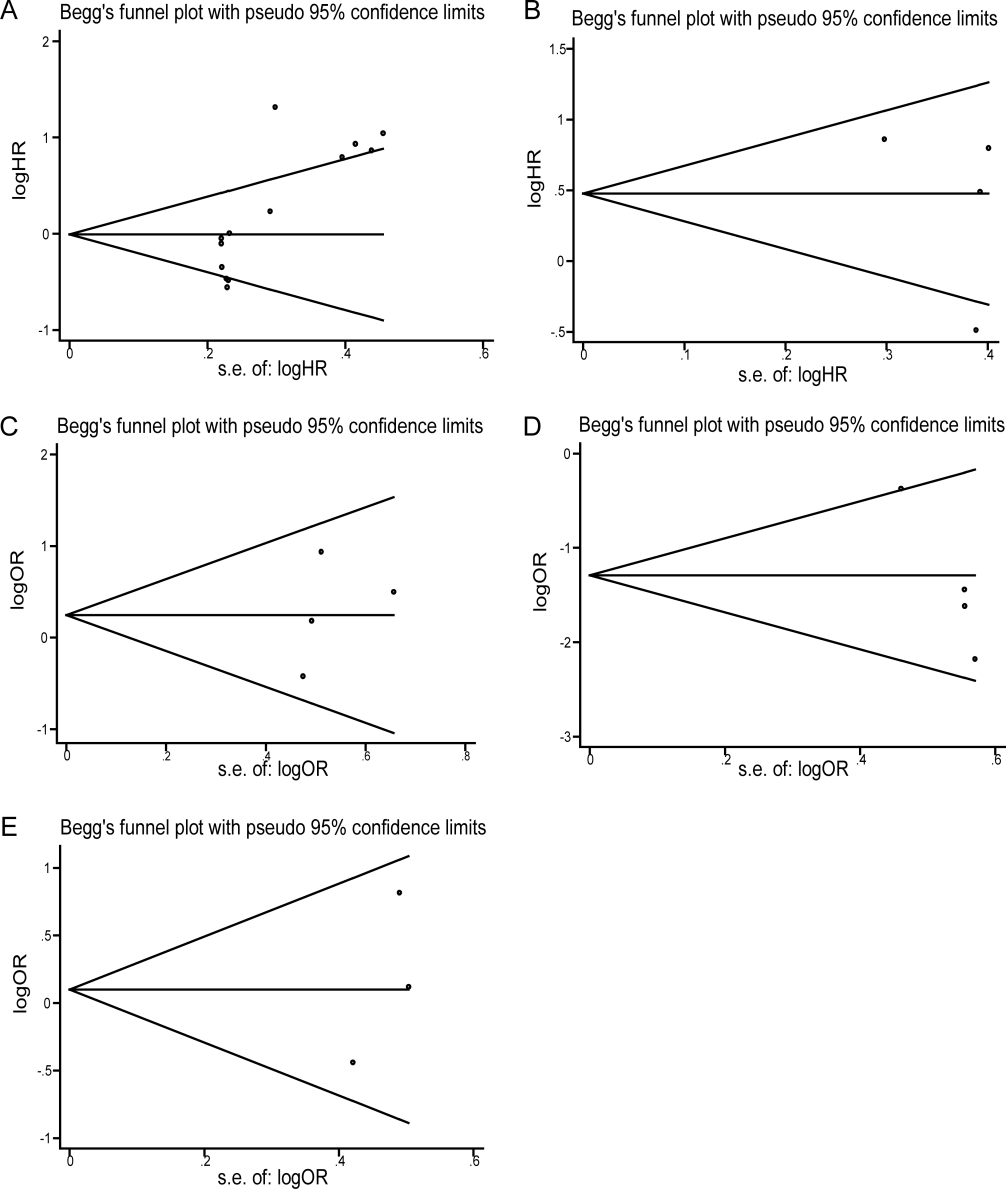
Funnel plot of the publication bias. (A) Funnel plot of the publication bias for overall survival. (B) Funnel plot of the publication bias for recurrence-free survival. (C) Funnel plot of the publication bias for gender. (D) Funnel plot of the publication bias for histological classifcation; (E) Funnel plot of the publication bias for multifocality.

Egger’s publication bias plot and Bgger’s funnel plot were performed to analyze the publication bias. Both the two tests indicated there were no publication bias, due to both the values of *P*>0.05. And the shape of funnel plots was approximately symmetrical (Fig 4).

### Sensitivity analysis

**Fig 5:**
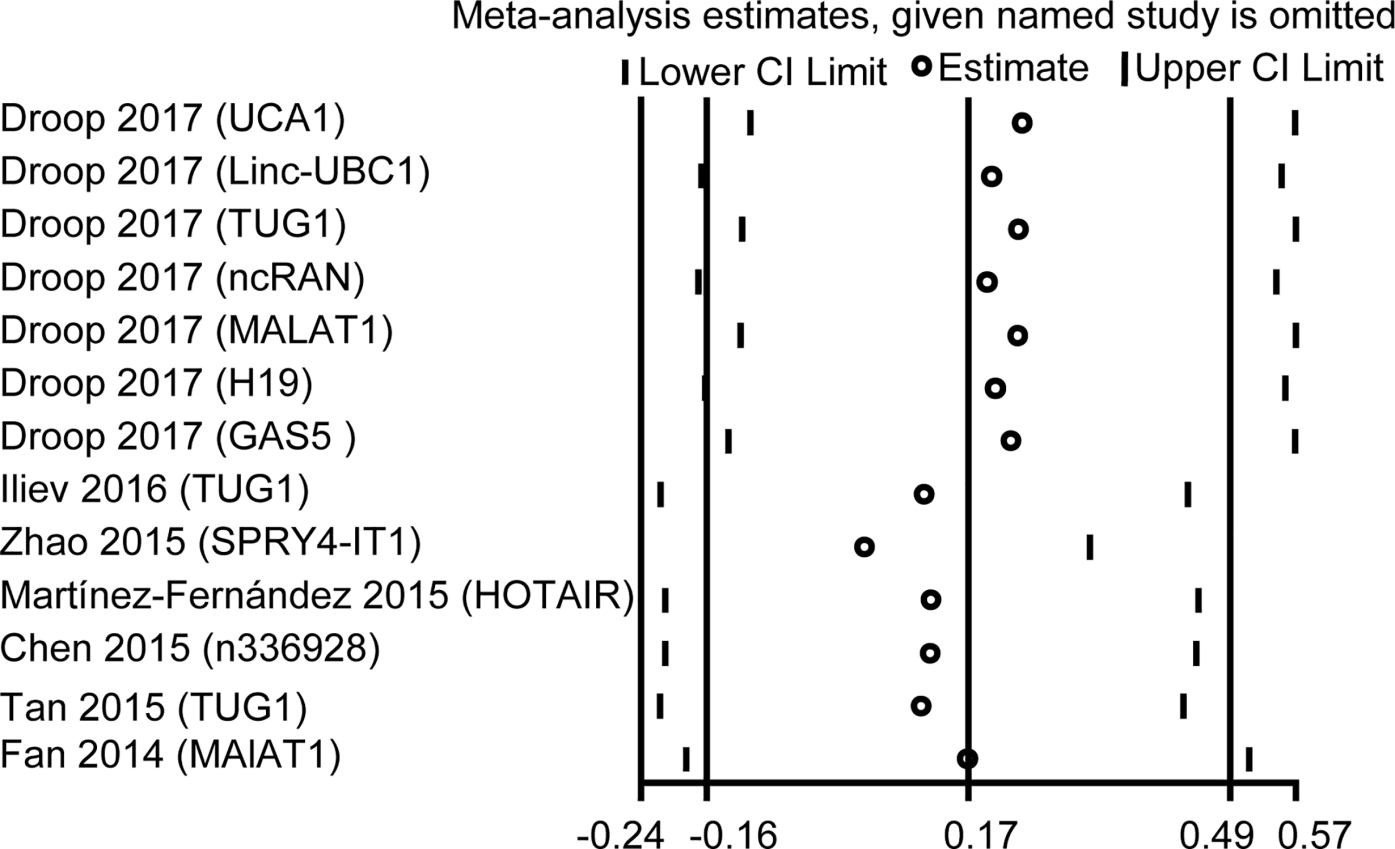
Sensitivity analysis of the effect of individual studies on the pooled HRs for lncRNAs expression and OS of BC patients.

Sensitivity analysis was performed to detect the influence of the individual study on the pooled results by removing one single study each time from the overall pooled analysis. The results verified that no individual study could change the pooled HRs significantly (Fig 5) and demonstrated that our analysis was relatively stable and credible.

## Discussion

Up to now, numerous researches have showed that lncRNAs are involved in various cell biological processes, including cellular differentiation, gene expression, protein localization, and DNA damage response. An increasing number of evidence revealed that aberrant expression of multiple lncRNAs was related to clinical outcomes for cancer patients. In order to find some prognostic biomarkers for BC, we conducted this comprehensive systematic meta-analysis of the current studies. The present meta-analysis is the first to systematically analyze the association between the expression of lncRNAs and BC prognosis.

In the present meta-analysis, we examined the prognostic role of lncRNAs in BC and the association between lncRNAs and clinicopathological characteristics. A total of 9 recently published articles comprising 13 lncRNAs and 666 BC patients were included into this meta-analysis. The combined HRs suggested that high lncRNAs transcription levels represent an independent OS factor among Asian patients with BC and their high expressions were associated with shorter OS. However, no obvious association was found in Caucasians. Racial classification and regional factors might be crucial in the prognosis of patients with BC. This might be related to the variations in life styles, ethnic genetic heterogeneity, etc. When grouped according to the follow-up period, we found that the association was significant for studies with follow-up period ≤ 60 months, indicating that the lncRNAs expression might be more valuable on predicting short-term outcome of BC. In addition, we explored the relation between lncRNAs expression and clinicopathological characteristics. We found that high lncRNAs expression was only significantly associated with Histological grade (G2-G3 vs. G1: OR = 3.857, 95%CI: 1.293–11.502, *P* = 0.015, random-effect).

Several researches have showed that the increased expression of 6 lncRNAs (H19[31], UCA1[32], TUG1 [33], MALAT1 [34], SPRY4-IT1 [35], and HOTAIR [36]) was correlated to poor prognostic outcome of cancers, those findings in consist with our results. And it has been reported that the lncRNAs were aberrantly expressed in a variety of cancers (Table 4), leading to lack of specific BC-related lncRNA. Therefore, identification of BC related lncRNAs that are vital in tumorigenesis are promising biomarkers for BC prognosis.

**Table 4:** LncRNAs were aberrantly expressed in a variety of cancers.

LncRNAs	Cancers
TUG1	NSCLC, BC, ESCC, Osteosarcoma, SCLC, CRC, ccRCC and GC
MEG3	NSCLC, GC, TSCC, NFPAs, HCC, osteosarcoma, PC and GC
MALAT1	NSCLC, HCC, GC, PDAC, CRC, ccRCC, BC, EC, Glioma, GBC, osteosarcoma and breast cancer
SPRY4-IT1	ccRCC, ESCC,BC, GC, glioma melanoma
HOTAIR	breast cancer, CRC,laryngeal squamous cell carcinoma, liver cancer, OC

NSCLC = non-small cell lung cancer; HCC = hepatocellular carcinoma; GC = gastric cancer; PDAC = pancreatic ductal adenocarcinoma; CRC = colorectal cancer; ccRCC = clear cell renal cell carcinoma; ESCC = esophageal squamous cell carcinoma; EC = esophageal cancer; GBC = gallbladder cancer; BC = bladder cancer; SCLC = small cell lung cancer; PC = prostate cancer; OC = ovarian cancer; GBC = gallbladder cancer; TSCC = tongue squamous cell carcinoma; NFPAs = non functioning pituitary adenomas

In the present study, lncRNAs(UNMIBC, MEG3, SNHG16, and Malat1) expression were not significantly associated with RFS. Unexpectedly, previous studies have found that low level of MEG3 lncRNA expression correlates with poor survival in multiple cancers[37] and patients with low MEG3 level had shorter recurrence-free survival (RFS) in bladder cancer[24]. Our meta-analysis has a obvious heterogeneity existed between studies. It is likely that the heterogeneity affect the pooled results. The sources of heterogeneity were diverse, such as tumour stages, molecular subtypes, analysis method and so on. However, due to the limited number of included articles, meta regression analysis, sensitivity analysis, and assessment of publication bias were not performed. So the results need to be confirmed by future studies with larger samples.

It should be stressed that there are several limitations in our meta-analysis. Firstly, we only included the studies that measured survival outcomes with OS and RFS, and the articles reporting other prognostic indicators were thus excluded; secondly, the number of studies included in our meta-analysis was inadequate and the sample size was limited; thirdly, age is a very important predictor of OS and RFS in bladder cancer[38]. Because of the included studies provided insufficient data and grouped according to different criteria, age of the BC patients could not be considered when evaluating the association of lncRNA expression with overall survival or clinical characteristics. To reach a definitive conclusion, further well-designed meta-analysis and high-quality studies are needed to confirm the association between the expression of lncRNAs and BC prognosis.

## Conclusion

In general, our meta-analysis for the first time evaluated the prognostic value of lncRNAs and the association between lncRNAs and clinical characteristics of patients with BC. Despite the existence of limitations, the present analysis showed that lncRNAs could be used as potential prognostic markers for BC and high lncRNAs expression could predict poor prognosis among Asian BC patients. We also found that lncRNAs could be developed as predictive biomarkers for Histological grade. However, in view of the limitation of individual studies about lncRNAs, good quality and large-scale investigations should be still warranted to further validate the clinical utilities of lncRNAs in evaluating BC patients’ prognosis.

## Supporting information

S1 Checklist PRISMA checklist. Each section was localized in the paper.
(DOC)

## References

[1] GLOBOCAN. Cancer incidence, mortality and prevalence worldwide in 2012 Lyon, France: International Agency Research on Cancer, 2012 Available from: http://globocan.iarc.fr

[2] Babjuk M, Burger M, Zigeuner R, Kaasinen E, Böhle A, Palou-Redorta J, et al EAU guidelines on non-muscle invasive urothelial carcinoma of the 2011 update. Eur Urol 2013;64:639–653. doi: 10.1016/j.eururo.2013.06.003 23827737 10.1016/j.eururo.2013.06.003

[3] Van Rhijn BW, Burger M, Lotan Y, Solsona E, Stief CG, Sylvester RJ, et al Recurrence and progression of disease in non-muscle-invasive bladder cancer: from epidemiology to treatment strategy. Eur Urol. 2009;56:430–442. doi: 10.1016/j.eururo.2009.06.028 19576682 10.1016/j.eururo.2009.06.028

[4] Kim WJ, Kim EJ, Kim SK, Kim YJ, Ha YS, Jeong P, et al Predictivevalue of progression-related gene classifier in primary non-muscle invasive bladder cancer. Mol Cancer. 2010;9:3 doi: 10.1186/1476-4598-9-3 20059769 10.1186/1476-4598-9-3 PMC2821358

[5] Kogo R, Shimamura T, Mimori K, Kawahara K, Imoto S, Sudo T, et al Long non-coding RNA HOTAIR regulates Polycomb-dependent chromatin modification and is associated with poor prognosis in colorectal cancers. Cancer Res. 2011;71:6320–6. doi: 10.1158/0008-5472.CAN-11-1021 21862635 10.1158/0008-5472.CAN-11-1021

[6] Maruyama Reo, Suzuki Hiromu. Long noncoding RNA involvement in cancer. BMB Reports. 2012;45 (11):604–611. doi: 10.5483/BMBRep.2012.45.11.227 23186998 10.5483/BMBRep.2012.45.11.227 PMC4133807

[7] Geng YJ, Xie SL, Li Q, Ma J, Wang GY. Large intervening non-coding RNA HOTAIR is associated with hepatocellular carcinoma progression. J Int Med Res [Internet]. 2011;39(6):2119–28. doi: 10.1177/147323001103900608 22289527 10.1177/147323001103900608

[8] Faghihi MA, Modarresi F, Khalil AM, Wood DE, Sahagan BG, Morgan TE, et al Expression of a noncoding RNA is elevated in Alzheimer’s disease and drives rapid feed-forward regulation of beta-secretase. Nat Med. 2008;14:723 doi: 10.1038/nm1784 18587408 10.1038/nm1784 PMC2826895

[9] Reis EM, Verjovski-Almeida S. Perspectives of Long Non-Coding RNAs in Cancer Diagnostics. Front Genet. 2012;3:32 doi: 10.3389/fgene.2012.00032 22408643 10.3389/fgene.2012.00032 PMC3297063

[10] Sana J, Faltejskova P, Svoboda M, Slaby O. Novel classes of noncoding RNAs and cancer. J Transl Med. 2012;10:103 doi: 10.1186/1479-5876-10-103 22613733 10.1186/1479-5876-10-103 PMC3434024

[11] Gutschner T, Diederichs S. The hallmarks of cancer: a long noncoding RNA point of view. RNA Biol. 2012;9:703–19. doi: 10.4161/rna.20481 22664915 10.4161/rna.20481 PMC3495743

[12] Qu Z, Yuan CH, Yin CQ, Guan Q, Chen H, Wang FB. Meta-analysis of the prognostic value of abnormally expressed lncRNAs in hepatocellularcarcinoma. Onco Targets Ther. 2016;9:5143–52. doi: 10.2147/OTT.S108599 27574455 10.2147/OTT.S108599 PMC4994879

[13] Wang M, Ma X, Zhu C, Guo L, Li Q, Liu M, et al The prognostic value of long non coding RNAs in non small cell lung cancer: A meta-analysis. Oncotarget. 2016;7(49):81292–81304. doi: 10.18632/oncotarget.13223 27833074 10.18632/oncotarget.13223 PMC5348393

[14] Jing W, Li N, Wang Y, Liu X1, Liao S1, Chai H, et al The prognostic significance of long noncoding RNAs in non-small cell lung cancer: a meta-analysis. Oncotarget. 2017; 8(3): 3957–3968. doi: 10.18632/oncotarget.13956 27992369 10.18632/oncotarget.13956 PMC5354806

[15] Yang Y, Wang S, Li T. Altered long non-coding RNAs predict worse outcome in osteosarcoma patients: evidencefrom a meta-analysis. Oncotarget. 2017; 8(21):35234–35243. doi: 10.18632/oncotarget.16470 28415638 10.18632/oncotarget.16470 PMC5471049

[16] Luo P, Liu XF, Wang YC, Li ND, Liao SJ, Yu MX, et al Prognostic value of abnormally expressed lncRNAs in ovarian carcinoma: a systematicreview and meta-analysis. Oncotarget. 2017;8(14):23927–23936. doi: 10.18632/oncotarget.14760 28118613 10.18632/oncotarget.14760 PMC5410355

[17] Chen J, Chen Y, Gu L, Li X, Gao Y, Lyu X, et al LncRNAs act as prognostic and diagnostic biomarkers in renal cell carcinoma: a systematic review and meta-analysis. Oncotarget. 2016; 7(45):74325–74336. doi: 10.18632/oncotarget.11101 27527868 10.18632/oncotarget.11101 PMC5342056

[18] Xue Y, Ma G, Zhang Z, Hua Q, Chu H, Tong N, et al: A novel antisense long noncoding RNA regulates the expression of MDC1 in bladder cancer. Oncotarget. 2015; 6: 484 doi: 10.18632/oncotarget.2861 25514464 10.18632/oncotarget.2861 PMC4381609

[19] Moher D, Liberati A, Tetzlaff J, Altman DG. Preferred reporting items for systematic reviews and meta-analyses: the PRISMA statement. J Clin Epidemiol. 2009;62:1006–1012. doi: 10.1016/j.jclinepi.2009.06.005 19631508 10.1016/j.jclinepi.2009.06.005

[20] Stang A. Critical evaluation of the Newcastle-Ottawa scale for the assessment of the quality of nonrandomized studies in meta-analyses. European journal of epidemiology. 2010; 25: 603–605. doi: 10.1007/s10654-010-9491-z 20652370 10.1007/s10654-010-9491-z

[21] Parmar MK, Torri V, Stewart L. Extracting summary statistics to perform meta-analyses of the published literature for survival endpoints. Stat Med. 1998;17:2815–34. 9921604 10.1002/(sici)1097-0258(19981230)17:24<2815::aid-sim110>3.0.co;2-8

[22] Droop J, Szarvas T, Schulz WA, Niedworok C, Niegisch G, Scheckenbach K, et al Diagnostic and prognostic value of long noncoding RNAs as biomarkers in urothelial carcinoma. PLoS One. 2017; 12(4): e0176287 doi: 10.1371/journal.pone.0176287 28430799 10.1371/journal.pone.0176287 PMC5400278

[23] Zhang S, Zhong G, He W, Yu H, Huang J, Lin T. lncRNA Up-Regulated in Nonmuscle Invasive Bladder Cancer Facilitates Tumor Growth andActs as a Negative Prognostic Factor of Recurrence. J Urol. 2016; 196(4):1270–8. doi: 10.1016/j.juro.2016.05.107 27267320 10.1016/j.juro.2016.05.107

[24] Duan W, Du L, Jiang X, Wang R, Yan S, Xie Y, et al Identification of a serum circulating lncRNA panel for the diagnosis and recurrence prediction of bladder cancer. Oncotarget. 2016;7:78850–58. doi: 10.18632/oncotarget.12880 27793008 10.18632/oncotarget.12880 PMC5346682

[25] Iliev R, Kleinova R, Juracek J, Dolezel J, Ozanova Z, Fedorko M. et al Overexpression of long non-coding RNA TUG1 predicts poor prognosis and promotes cancer cell proliferation and migration in high-grade muscle-invasive bladder cancer. Tumour Biol. 2016; 37: 13385–13390. doi: 10.1007/s13277-016-5177-9 27460088 10.1007/s13277-016-5177-9

[26] Zhao XL, Zhao ZH, Xu WC, Hou JQ, Du XY. Increased expression of SPRY4-IT1 predicts poor prognosis and promotes tumor growth and metastasis in bladder cancer. Int J Clin Exp Pathol. 2015;8:1954–1960. 25973088 PMC4396312

[27] Martínez-Fernández M, Feber A, Dueñas M, Segovia C, Rubio C, Fernandez M, et al Analysis of the Polycomb-related lncRNAs HOTAIR and ANRIL in bladder cancer. Clin Epigenetics. 2015;7:109 doi: 10.1186/s13148-015-0141-x 26457124 10.1186/s13148-015-0141-x PMC4599691

[28] Chen T, Xie W, Xie L, Sun Y, Zhang Y, Shen Z, et al Expression of long noncoding RNA lncRNA-n336928 is correlated with tumor stage and grade and overall survival in bladder cancer. Biochem Biophys Res Commun. 2015;468:666–70. doi: 10.1016/j.bbrc.2015.11.013 26551459 10.1016/j.bbrc.2015.11.013

[29] Tan J, Qiu K, Li M, Liang Y. Double-negative feedback loop between long non-coding RNA TUG1 and miR-145 promotes epithelial to mesenchymal transition and radioresistance in human bladder cancer cells. FEBS Lett. 2015; 589:3175–81. doi: 10.1016/j.febslet.2015.08.020 26318860 10.1016/j.febslet.2015.08.020

[30] Fan Y, Shen B, Tan M, Mu X, Qin Y, Zhang F, et al TGF-beta-induced upregulation of malat1 promotes bladder cancer metastasis by associating with suz12. Clin Cancer Res. 2014;20:1531–1541. doi: 10.1158/1078-0432.CCR-13-1455 24449823 10.1158/1078-0432.CCR-13-1455

[31] Chen T, Yang P, He Z. Long non-coding RNA H19 can predict a poor prognosis and lymph node metastasis: a meta-analysis in human cancer. Minerva Med. 2016;107(4):251–258. 27348443

[32] Li J, Gao J, Kan A, Hao T, Huang L. SNHG and UCA1 as prognostic molecular biomarkers in hepatocellular carcinoma: recent research and meta-analysis. Minerva Med. 2017;108(6):568–574. doi: 10.23736/S0026-4806.17.05094-7 28466631 10.23736/S0026-4806.17.05094-7

[33] Zhou Y, Lu Y, Li R, Yan N, Li X, Dai T. Prognostic role of long non-coding RNA TUG1 expression in various cancers: a meta-analysis. Oncotarget. 2017;8:100499–100507. doi: 10.18632/oncotarget.20037 29245996 10.18632/oncotarget.20037 PMC5725038

[34] Shuai P, Zhou Y, Gong B, Jiang Z, Yang C, Yang H, et al Long noncoding RNA MALAT1 can serve as a valuable biomarker for prognosis and lymph node metastasis in various cancers: a meta-analysis. Springerplus. 2016;5(1):1721 doi: 10.1186/s40064-016-3342-7 27777857 10.1186/s40064-016-3342-7 PMC5052238

[35] Li N, Tan Q, Jing W, Luo P, Tu J. Long Non-Coding RNA SPRY4-IT1 Can Predict Unfavorable Prognosis and Lymph Node Metastasis: a Meta-Analysis. Pathol Oncol Res. 2017; 23(4):731–736. doi: 10.1007/s12253-016-0182-2 28054316 10.1007/s12253-016-0182-2

[36] Xi W, Song W. Prognostic value of lncRNA HOTAIR expression in patients with cancer: A Meta-analysis. Zhong Nan Da Xue Xue Bao Yi Xue Ban. 2016;41(12):1352–1357. doi: 10.11817/j.issn.1672-7347.2016.12.017 28070051 10.11817/j.issn.1672-7347.2016.12.017

[37] Cui X, Jing X, Long C, Tian J, Zhu J. Long noncoding RNA MEG3, a potential novel biomarker to predict the clinical outcome of cancer patients: a meta-analysis. Oncotarget. 2017; 8(12): 19049–19056. doi: 10.18632/oncotarget.14987 28157702 10.18632/oncotarget.14987 PMC5386668

[38] Kluth LA, Black PC, Bochner BH, et al Prognostic and prediction tools in bladder cancer: a comprehensive review of the literature. Eur Urol 2015;68:238–53. doi: 10.1016/j.eururo.2015.01.032 25709027 10.1016/j.eururo.2015.01.032

