# Expression of Concern: The prognostic significance of long noncoding RNAs in bladder cancer: A meta-analysis

**DOI:** 10.1371/journal.pone.0284938

**Published:** 2023-04-24

**Authors:** The PLOS ONE Editors

After this article was published, similarities were noted between this article [1] and submissions by other research groups which call into question the validity and provenance of the reported results.

In response to queries about these concerns, the first author provided the underlying data in S1–S4 Files. They also noted that there are errors in:

- Fig 4D, where there is an additional data point (three articles were analyzed but there are four data points in Fig 4D). An updated Fig 4D with three data points is provided below.
- Table 1, where the HR value for ref. 22 UCA1 should be 0.567.

**Fig 4:**
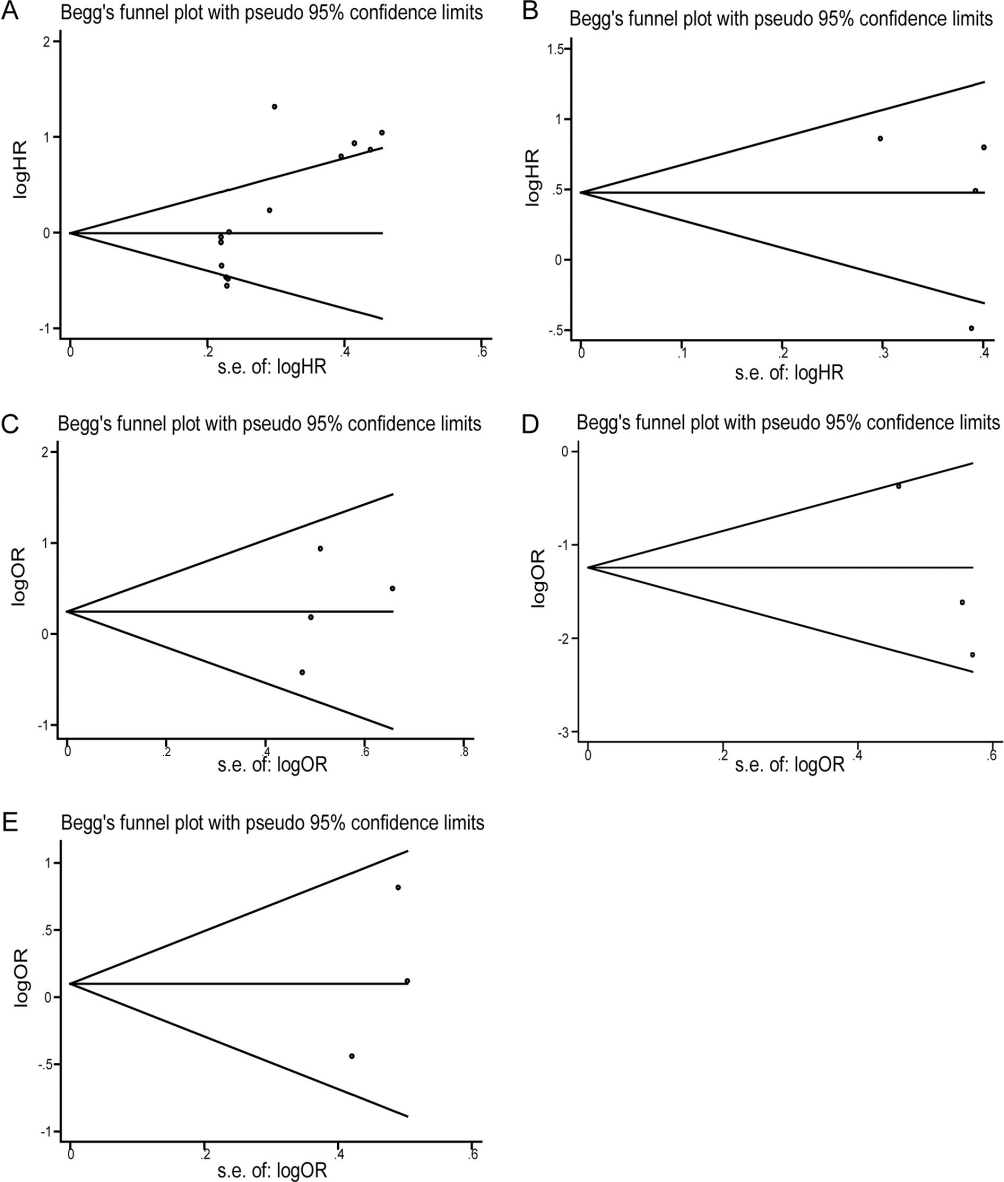
Funnel plot of the publication bias. (A) Funnel plot of the publication bias for overall survival. (B) Funnel plot of the publication bias for recurrence-free survival. (C) Funnel plot of the publication bias for gender. (D) Funnel plot of the publication bias for histological classification; (E) Funnel plot of the publication bias for multifocality.

The authors commented on aspects of how data were collected and analyzed for this study. The *PLOS ONE* Editors concluded that the data and information provided in post-publication discussions lent some assurance as to the results reported in the article, but did not address the concerns about similarities with other published and submitted work.

The *PLOS ONE* Editors issue this Expression of Concern to notify readers of the unresolved concerns discussed above, and to provide the data and updated figure received from the authors.

The Director of Cell Biology at Dalian Medical University informed PLOS that the University investigated the primary research records and confirmed that this article represents the authors’ work.

The Competing Interests statements has been updated to: Author ZL is affiliated with Biomedical Business Department, Panasonic Appliances Cold Chain (Dalian) co., Ltd.

## Supporting information

S1 File The underlying data used for the analysis in this article [1]. (RAR)

S2 File Individual-level quantitative data underlying Fig 4. (XLSX)

S3 File Individual-level quantitative data underlying Fig 5. (XLSX)

S4 File A list of excluded articles and reasons for exclusion from the analysis in this article [1]. (XLSX)
